# Biochemical and molecular analysis in mucopolysaccharidoses: what a paediatrician must know

**DOI:** 10.1186/s13052-018-0553-2

**Published:** 2018-11-16

**Authors:** Mirella Filocamo, Rosella Tomanin, Francesca Bertola, Amelia Morrone

**Affiliations:** 1 Laboratorio di Genetica Molecolare e Biobanche, Istituto G. Gaslini, Genova, Italy; 2 Laboratory of Diagnosis and Therapy of Lysosomal Disorders, Department of Women’s and Children’s Health, University of Padova, Padova, Italy; 3 School of Medicine and Surgery, University of Milano Bicocca, Monza, Italy; 4 Neuroscience Department, Molecular and Cell Biology Laboratory of Neurometabolic Diseases, Meyer Children’s Hospital, University of Florence, Florence, Italy; 5 Department of Neurofarba, University of Florence, Florence, Italy

**Keywords:** Laboratory tests, Mucopolysaccharides, Glycosaminoglycans, Molecular analysis, Pseudodeficiency, Genetic counselling, Genotype-phenotype relationship, Lysosomal storage disorders

## Abstract

Mucopolysaccharidoses (MPS) are rare inherited disorders caused by a deficit of the lysosomal hydrolases involved in the degradation of mucopolysaccharides, also known as glycosaminoglycans (GAGs). They are all monogenic defects, transmitted in an autosomal recessive way, except for MPS type II which is X-linked. The enzymatic deficit causes a pathologic accumulation of undegraded or partially degraded substrates inside lysosomes as well as in the extracellular compartment. MPS generally present with recognizable signs and symptoms to raise a clinical suspicion. However, although they have individual peculiarities, often signs and symptoms may overlap between different MPS types. Therefore, a deeper evaluation of specific disease biomarkers becomes necessary to reach an appropriate diagnosis. This paper stresses the central role of the laboratory in completing and confirming the clinical suspicion of MPS according to a standardized procedure: first, a biochemical evaluation of the patient samples, including qualitative/quantitative urinary GAG analysis and a determination of enzyme activities, and then the molecular diagnosis. We also encourage a constant and close communication between clinicians and laboratory personnel to address a correct and early MPS diagnosis.

## Background

The degradation of the glycosaminoglycans (GAGs or mucopolysaccharides), a major component of the extracellular matrix, joint fluid, and connective tissue, takes place in the lysosomes. Under physiological conditions, the main GAG chains — dermatan sulphate (DS), heparan sulphate (HS), keratan sulphate (KS), and chondroitin sulphate (CS) — are degraded by 11 lysosomal hydrolases through the sequential removal of monosaccharides followed by the removal of sulphate groups, resulting in the complete degradation of the polysaccharide to its individual components.

**Fig. 1:**
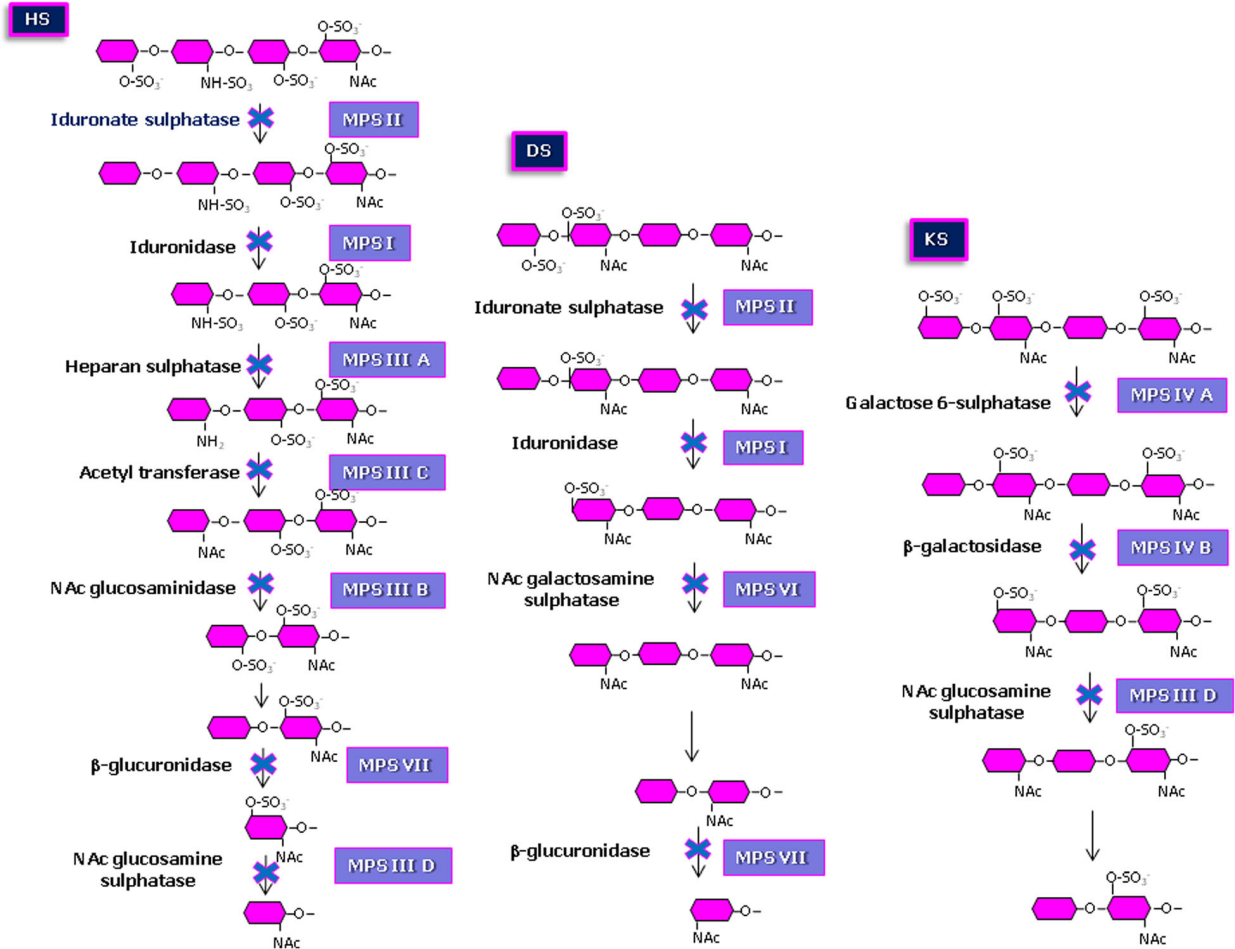
Stepwise degradation of the main glycosaminoglycan chains, heparan sulphate (HS), dermatan sulphate (DS), and keratan sulphate (KS). The enzymes involved in the pathway are shown in black. Defective enzyme activity leading to the different types of mucopolysaccharidosis (MPS) is indicated in blue. Note that the degradation pathway of chondroitin sulphates, being similar to that of DS, is not shown

The deficit of any one of the 11 acid hydrolase activities gives rise to the progressive accumulation of GAGs in most tissues and organ systems, as well as in urine. Figure 1 illustrates the stepwise degradation of the main GAG chains by specific enzymes, as well as the resulting 11 distinct types of mucopolysaccharidoses (MPS) depending on the enzyme deficiency. Table 1 summarises the various enzyme defects and the type(s) of accumulated GAGs for each specific type/subtype of MPS. The 11 genes involved in the different subgroups of MPS have already been identified and characterized (Table 2), as well as the hereditary transmission model: all MPS have an autosomal recessive transmission with the exception of MPS II, which exhibits an X-linked pattern.

**Table 1:** Classification and major characteristics of the mucopolysaccharidoses (MPS)

Type	Syndrome	OMIM #	Enzyme defect	Affected GAG
MPS IH	Hurler	607014	α-l-iduronidase	DS, HS
MPS IS	Scheie	607,015		
MPS IH/S	Hurler-Scheie	607016		
MPS II	Hunter (severe)	309900	Iduronate 2-sulfatase	DS, HS
	Hunter (attenuated)			
MPS IIIA	Sanfilippo A	252900	Heparan *N*-sulfatase	HS
MPS IIIB	Sanfilippo B	252920	α-*N*-acetyl-d-glucosaminidase	
MPS IIIC	Sanfilippo C	252930	Acetyl CoA:α-glucosaminide-acetyltransferase	
MPS IIID	Sanfilippo D	252940	*N*-acetylglucosamine-6-sulfatase	
MPS IVA	Morquio A	253000	*N*-acetylgalactosamine-6-sulfatase	KS, CS
MPS IVB	Morquio B	253010	β-galactosidase	KS
MPS VI	Maroteaux-Lamy	253200	*N*-acetylgalactosamine-4-sulfphatase (arylsulphatase B)	DS
MPS VII	Sly	253220	β-glucuronidase	DS, HS, CS
MPS IX	–	601492	Hyaluronidase	Hyluronan

MPS V and VIII are designations no longer used

*CS* chondroitin sulphate, *DS* dermatan sulphate, *GAG* glycosaminoglycan, *HS* heparan sulphate, *KS* Keratan sulphate

**Table 2:** Characteristics and types of mutations of the 11 genes responsible for the different subgroups of the mucopolysaccharidoses (MPS)^a^

	MPS IH/S	MPS II	MPS IIIA	MPS IIIB	MPS IIIC	MPS IIID	MPS IVA	MPS IVB	MPS VI	MPS VII	MPS IX
Gene											
Name	α-l-Iduronidase	Iduronate 2-sulphatase	Heparan-*N*- sulphatase	α-N-acetyl-d-glucosaminidase	Heparan-α-glucosaminide *N*-acetyltransferase	Glucosamine (*N*-acetyl)-6-sulphatase	Galactosamine (*N*-acetyl)-6-sulphate sulphatase	β-Galactosi-dase	Aryl-sulphatase B	β-glucuroni-dase	Hyalurono-glucosami-nidase 1
Gene symbol	*IDUA*	*IDS*	*SGSH*	*NAGLU*	*HGSNAT*	*GNS*	*GALNS*	*GLB1*	*ARSB*	*GUSB*	*HYAL1*
OMIM #	252800	300823	605270	609701	610453	607664	612222	611458	611542	611499	607071
Chr locus	4p16.3	Xq28	17q25.3	17q21	8p11.1	12q14	16q24.3	3p21.33	5q11-q13	7q21.11	3p21.3-p21.2
Mutation type											
Missense/nonsense	148	313	111	114	38	7	248	165	147	53	1
Regulatory	1	0	0	0	0	0	0	0	0	1	0
Splicing	39	59	3	6	14	4	32	16	11	5	0
Small deletions (< 21 bp)	40	117	17	25	5	5	32	17	24	4	1
Small insertions (< 21 bp)	16	49	9	13	5	4	5	12	6	0	0
Small indels	3	14	1	1	1	1	2	2	2	0	0
Gross deletions (> 20 bp)	6	52	3	4	3	2	9	1	6	1	0
Gross insertions (> 20 bp)	1	4	1	3	1	0	2	2	0	0	0
Complex rearrangements	3	20	0	0	1	2	3	0	0	0	1
Total	257	628	145	166	68	25	333	215	197	64	3

^a^Data were obtained from the Human Gene Mutation Database Professional (release HGMD® Professional 2017.3). See http://www.hgmd.org

### Laboratory diagnosis

#### Biochemical assay

Preliminary diagnostic analyses range from semiquantitative spot tests [1] to quantitative assays [2, 3] followed by qualitative identification [4] of increased urinary GAGs.

It should be noted that none of these methods can be considered diagnostic per se since false negative/positive results are sometimes obtained. A relatively high incidence of false negative urinary screening has been reported for HS in MPS III patients with mild/intermediate clinical phenotypes [5] (Filocamo, personal data). KS does not form a reaction product with any of these routinely used methods; hence, quantitative GAG assessment in Morquio syndrome (MPS IV) is unreliable. An enzyme-linked immunosorbent assay (ELISA) technique has been shown to quantify KS accurately in the urine and blood of patients with Morquio syndrome type A [6]. It is also known that several factors can interfere with the measurement of urinary GAGs and lead to false positive results. For instance, the presence of the anticoagulant heparin, and blood or haemoglobin, as well as several diseases other than MPS (leukaemia, rheumatic arthritis, diabetes, obesity, etc.), may also lead to an abnormally high GAG excretion in the urine [7, 8].

Moreover, assays using tandem mass spectrometry (MS/MS) have been established to measure GAG levels in serum or plasma and, more recently, on dried blood spot (DBS). MS/MS, allowing for the simultaneous measurement of several GAGs, provides a sensitive, specific, and reproducible GAG analysis making it potentially useful for the screening, prognosis, and monitoring of any therapeutic effect in MPS patients [9, 10].

#### Enzymatic assays

The first step in the diagnostic process includes specific enzymatic assays in a variety of cells. Leukocytes, cultured lymphoblasts, or fibroblasts are generally used, with the actual choice depending upon the characteristics of the enzyme to be assessed as well as the corresponding method of detection. Enzyme activities are generally determined by fluorogenic (4-methylumbelliferyl) or, rarely, chromogenic (p-nitrophenyl) substrates.

##### Dried blood spot (DBS)

DBS testing is a good option for new-born screening (NBS) for various lysosomal storage disorders including some MPS [11–13].

Although this approach is helpful for certain disorders, positive DBS results need to be validated by conventional testing, in other words enzymatic assay in cellular extracts and/or molecular analysis. It is important for clinicians to understand how to interpret results from DBS screening as in the near future it is likely that NBS programmes will include DBS tests. For comprehensive reviews on this topic the reader can consult a relevant chapter in this same supplement.

##### Pseudodeficiency

Generally, a deficient activity of one of the 11 lysosomal enzymes involved in GAG degradation is associated with an MPS. However, there are individuals who show greatly reduced enzymatic activity but remain clinically healthy. This condition, termed enzymatic pseudodeficiency (Pd), is due to polymorphic genetic variants and can affect some of these lysosomal hydrolases. The potential presence of an enzymatic Pd poses a limitation for enzymatic tests and should be investigated whenever the results from an enzymatic assay do not concur with the clinical phenotype of the patient. The currently known enzymatic pseudodeficiencies are reported below, on a disease-by-disease basis.

#### Molecular analysis

Molecular testing, available for all MPS, follows the enzymatic diagnosis and serves to refine the diagnosis; it can also be extremely helpful in facilitating familial genetic counselling. In fact, once the genotype of an individual affected patient has been ascertained, genetic counselling should address the possible prediction of the potential clinical phenotype, indications for the most appropriate therapy, if any, and the prospect for identification of any at-risk carriers in the family.

**Fig. 2:**
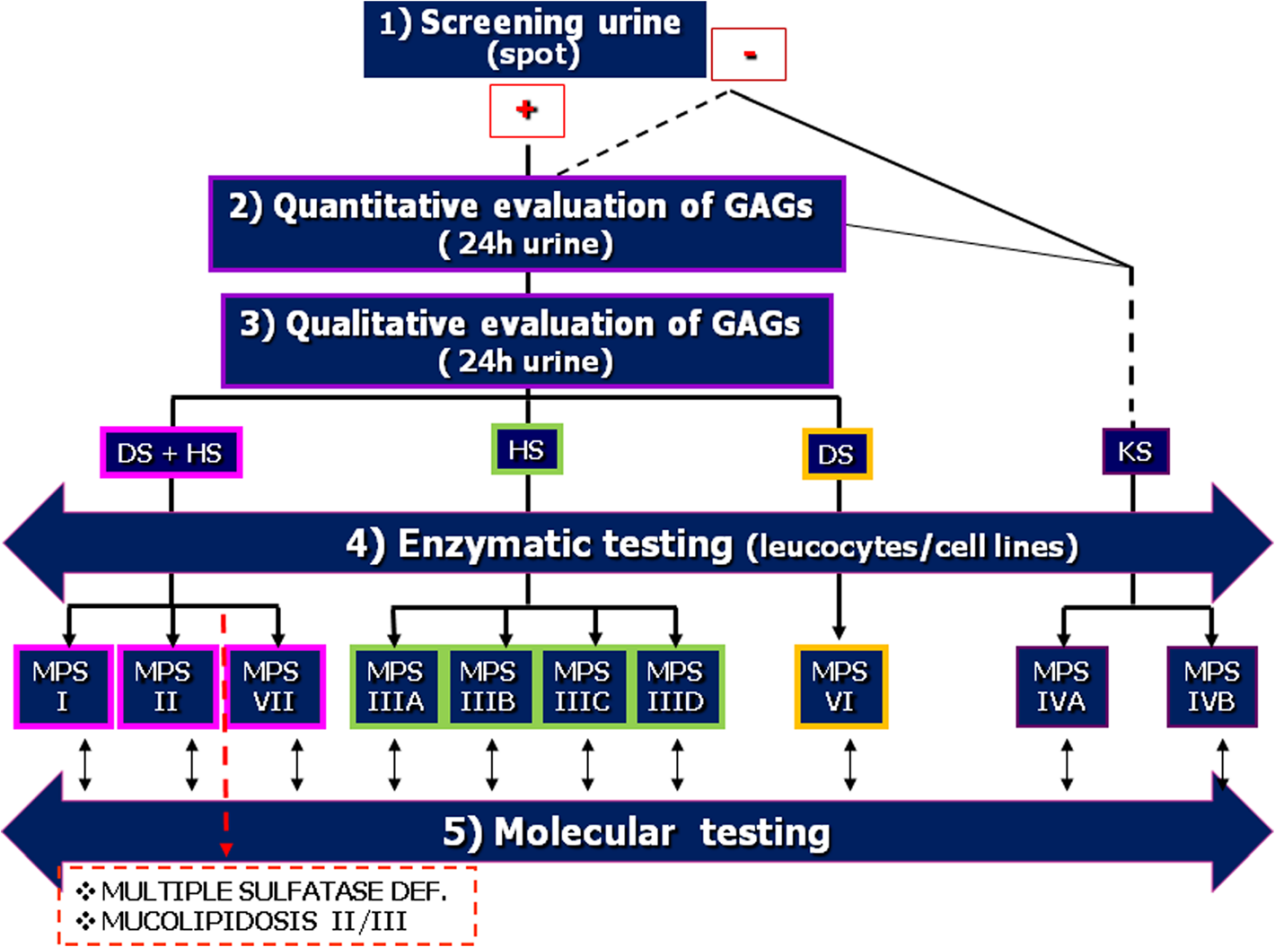
Diagnostic flow chart for mucopolysaccharidoses (MPS). The diagnosis of the MPS can be performed starting with a screening of the urine followed by quantitative assay of urinary glycosaminoglycans (GAGs) and, if available, by qualitative evaluation of the GAGs accumulated that can steer the enzymatic assay. Once the enzymatic defect has been determined, the molecular analysis will serve to identify the causative genomic variant. Note that keratan sulphate (KS) does not form a reaction product with any of the routinely methods reported here; hence, quantitative GAG assessment in Morquio syndrome (MPS IV) requires other techniques or the two enzymatic assays. Differential laboratory diagnosis includes multiple sulphatase deficiency and mucolipidosis II and III, in which GAGs accumulate because of their primitive defect involving some of the enzymes of the GAG degradation pathway. HS heparan sulphate, DS dermatan sulphate

Figure 2 graphically represents the conventional diagnostic flowchart.

##### Next-generation sequencing

The use of massively parallel sequencing analysis, known as next-generation sequencing (NGS), is becoming increasingly common as a fast and low-cost sequencing approach. It includes whole-genome sequencing (WGS), whole-exome sequencing (WES), and the so-called “targeted resequencing approach” with focused “gene panels” which are designed to include genomic regions known to underlie diseases grouped by clinical affinity. In the near future, it is possible that gene analysis using NGS techniques will replace preliminary tests based on enzyme activities or metabolite concentrations. This would offer several advantages, mainly in terms of speeding up the diagnosis in patients where a differential analysis is difficult to perform.

However, the NGS-based technology is not without limitations. It should be taken into consideration that this technology is “error-prone” and that the presence of variants of unknown significance (VUS) can make data analysis extremely complicated [14, 15]. Nor should we overlook the ethical issue that could arise because of potential incidental findings obtained from NGS-based technology.

Finally, it should be stressed that, for orphan diseases, it is often difficult to build a base of molecular knowledge as mutations tend to be private and the molecular classification of each novel variant necessarily implies additional laboratory investigations such as functional studies based on enzyme and metabolite assays, which are both cumbersome and costly. Without such a validation, it is hard to decide whether a new variant is disease-causing or benign and would risk falling into the VUS category.

##### Genetic counselling

All MPS are inherited as autosomal recessive disorders, except MPS II which is X-linked and therefore only affects males except in extremely rare cases. Once a definitive diagnosis is achieved, genetic counselling can be offered to patients and their families. Such counselling should explain the mode of inheritance and the importance of testing at-risk family members, it should yield an estimate of recurrence risks, and provide information about prenatal genetic tests and available therapeutic approaches.

Prenatal diagnosis is performed on the most appropriate samples, which usually include fresh or cultured chorionic villus samples obtained by villocentesis, usually performed at approximately 12 weeks gestation, or cultured amniotic fluid obtained via amniocentesis at approximately 16 weeks gestation. The choice of the test, enzymatic and/or molecular, is based on the characteristics of the defect to be investigated. Prenatal molecular testing requires the genotype of the family index case to be known. The availability of both tests (enzymatic and molecular) enormously increases the reliability of the diagnosis*.* Hence, biochemical and molecular genetic testing are usually complementary for the prenatal diagnosis of MPS. However, special considerations are required for the prenatal diagnosis of Hunter syndrome, the only X-linked MPS. Since in a female carrier a skewed distribution of the cells expressing the mutant allele might yield results resembling those of an affected male fetus, the usual procedure is to determine fetal sex and only if the karyotype is 46,XY would fetal cells be biochemically analysed antenatally.

##### Biobanking

Since our understanding of genes, mutations, and diseases is continually improving, consideration should be given to biobanking samples and data from affected individuals, as well as from their parents and first-degree relatives, for future diagnostic and research purposes [16].

More comprehensive information on diagnosis and management of individual MPS is provided in some recently published guidelines [17–22].

### Molecular genetics: Trying to understand the genotype-phenotype relationship

The elucidation of the molecular mechanisms underlying the MPS began in the 1990s. The process of cloning the diverse genes encoding the lysosomal enzymes involved in the GAG degradative pathways lasted until 2006 with the identification of the gene responsible for MPS IIIC. Although the large variety of mutations responsible for any MPS has often hampered the establishment of clear genotype–phenotype correlations, some general conclusions can be drawn in relation to recurrent mutations and are usually based on prior observations in large series of patients. However, even in the presence of a recurrent mutation, some patients carrying the same molecular lesion may present with different clinical phenotypes, suggesting that other variants at other gene loci and/or some environmental factors can modulate the clinical phenotype. This may be particularly important in the case of parents’ consanguinity, which is quite common in some ethnic groups due to geographical reasons or to the practice of arranged marriages [23–25].

Table 2 summarises the characteristics and types of mutations of the 11 genes responsible for the different MPS, as reported in the Human Gene Mutation Database [26].

#### MPS I

The gene encoding α-l-iduronidase (*IDUA*; MIM #252800) maps to chromosome 4p16.3 and contains 14 exons encoding for a polypeptide of 653 amino acids [27]. A deficiency of this enzyme results in the lack of degradation of DS and HS and their progressive accumulation. To date, at least 257 *IDUA* mutations are known, spread along the entire coding and splicing regions (Table 2). MPS I is a highly heterogeneous disorder with a wide spectrum of clinical manifestations [28, 29]. Overall, most mutations are ‘private’ with very few pan-ethnic mutations, the two most common being the p.Trp402X and p.Gln70X mutations found to be associated with a severe phenotype, and the p.Pro533Arg associated with an intermediate-severe phenotype; the p.Gly51Asp and p.Pro496Arg mutations have been found until now only in Italian patients, associated with a severe phenotype [30].

MPS I diagnosis can be hampered by the occurrence of α-l-iduronidase Pd. Although only the variant p.Ala300Thr has been reported so far as being associated with enzymatic Pd [31], other variants (p.Ala79Thr, p.His82Gln, p.Asp223Asn, and p.Val322Glu) are under investigation as candidates for Pd [32, 33]. Therefore, as NBS for MPS I has begun in some countries, it is imperative to follow-up a real deficient α-l-iduronidase result with urinary GAG analysis and *IDUA* gene sequencing prior to initiating treatment for MPS I.

#### MPS II

The iduronate 2-sulfatase gene (*IDS*; MIM #300823) contains nine exons and spans ~ 24 kb on Xq28. The 1650 bp open reading frame of the *IDS* gene is predicted to encode a 550-amino acid polypeptide [34]. The enzyme cleaves the sulphate group in position 2 of HS and DS; thus, its deficit results in the diffused pathological deposit of the both GAGs in most body districts. No cases with significant residual enzymatic activity have so far been described. A pseudogene, *IDS2*, presenting more than 88% homology with *IDS* exons 2 and 3, as well as introns 2, 3, and 7, is located 20 kb telomeric to the functional gene but in the opposite orientation [34]. The presence of the pseudogene renders molecular diagnosis more difficult to perform, since appropriate primers need to be designed to selectively avoid amplification of the homologous pseudogene sequences. To date, 628 different mutations in the *IDS* gene have been reported (Table 2). Among these, gross alterations including insertions, deletions, and gene-pseudogene recombinational rearrangements represent about 12% (76/628) of all known *IDS* gene lesions, 29% are small deletions, insertions and indels and 9% (59/628) are splicing mutations, while about 50% (313/628) are missense/nonsense variants. The *de novo* occurrence of *IDS* gene mutations has sometimes been demonstrated by family studies in large MPS II cohorts. Whilst germline mosaicism has been assumed in some cases, this has only rarely been formally demonstrated [35]. No high-frequency recurring mutations have been reported which has tended to constrain studies of the genotype-phenotype relationship. However, an approximate relationship may be based on the observation that, while missense variations may be associated with both severe and attenuated phenotypes, nonsense variants, splicing transcriptional defects, gross rearrangements, or deletions/insertions are more commonly associated with severe phenotypes. A recent study conducted on 65 Japanese families showed that missense variants were mainly associated with attenuated phenotypes (87.5%) and less frequently with severe phenotypes (29.3%) [36]. Additionally, identical mutations have been found in patients with both mild and severe disease, implying the possible contribution of other genetic or environmental modifiers on the phenotype [35, 37, 38]. Rare female MPS II patients have also been reported, mostly due to the non-random inactivation of the X chromosome (XCI) [27, 39–42]. Very rarely, a female MPS II patient may be due to a balanced reciprocal translocation involving one X chromosome, coupled with a skewed XCI of the normal X chromosome [43].

#### MPS IIIA

The HS sulphatase gene (*SGSH*; MIM #605270) spanning about 11 kb in length, comprises 8 exons. The most prominent of the three demonstrated transcripts (3.1, 4.3, and 7.1 kb) in most tissues, that of 3.1 kb, encodes a 502-amino acid sulphamidase protein [44]. The deficit of the heparan N-sulphatase enzyme results in the accumulation of the HS in organs and tissues. To date, of the 145 mutations reported in Table 2, only general conclusions have been drawn for p.Arg245His, p.Gln380Arg, p.Ser66Trp, and p.Val361SerfsX52 mutations, which have been found to be related to the development of a severe phenotype, while p.Gly122Arg, p.Arg206Pro, p.Ser298Pro, p.Ile322Ser, and p.Glu369Lys mutations have been reported as being associated with an attenuated phenotype [45]. Overall, no pan-ethnic recurrent mutations have been reported, although some mutations predominate in different ethnogeographic groups, reflecting possible founder effects. In particular, p.Arg74Cys and p.Val361SerfsX52 are frequent in Poland (56%) and Spain (45%), p.Arg245His in Germany (35%) and Holland (57%), and p.Ser66Trp in Italy (29%) [46–48].

#### MPS IIIB

The gene encoding α-*N*-acetyl-d-glucosaminidase (*NAGLU*; MIM #609701) is approximately 8.5 kb in length and comprises 6 exons. Its transcript of 2.7 kb encodes a protein of 743 amino acids [49, 50]. The defect of α-*N*-acetyl-d-glucosaminidase enzyme causes the accumulation of the HS in organs and tissues. A total of 166 *NAGLU* mutations have been described (Table 2). All the reported mutations occur at low frequencies, indicating an extensive *NAGLU* mutational heterogeneity which is likely to be responsible for the wide clinical spectrum of MPS IIIB. Some predictions have been made for p.Phe48Lys, p.Gly69Ser, p.Ser612Gly, and p.Arg643Cys mutations that appear to be related to a less severe clinical phenotype [51]. However, common mutations have not been identified in MPS IIIB patients.

A potential complication reported in MPS IIIB is the simultaneous presence of the two polymorphisms p.Ser141Ser and p.Arg737Gly producing a pseudodeficiency allele that leads to a reduced level of α-*N*-acetyl-d-glucosaminidase activity (Filocamo, personal data).

#### MPS IIIC

The heparin acetyl CoA:α-glucosaminide-*N*-acetyltransferase gene (*HGSNAT*; MIM #610453) contains 18 exons generating a 1908-base pair cDNA that encodes a 635-amino acid protein. The deficit of acetyl CoA:α-glucosaminide-acetyltransferase leads to the accumulation of HS in organs and tissues. To date, 68 mutations have been described for the *HGSNAT* gene (Table 2). Only two mutations (p.Arg344Cys and p.Ser518Phe) have been reported with a higher frequency in the Dutch population [52]. Despite the identification of several MPS IIIC-causing mutations, it has proven difficult to establish a clear genotype-phenotype correlation, except in a few cases such as mutations p.Gly262Arg and p.Ser539Cys from two patients that were associated with an attenuated phenotype [53, 54].

#### MPS IIID

The α-*N*-acetylglucosamine-6-sulphate sulphatase gene (*GNS*; MIM #607664) consists of 14 exons spanning approximately 46 kb of DNA; the predicted protein has 552 amino acids [55, 56]. The defect of *N*-acetylglucosamine-6-sulphatase causes the accumulation of HS in organs and tissues. Only 25, mostly private, disease-causing mutations in MPS IIID patients have been reported to date, thereby hampering genotype-phenotype correlation studies (Table 2).

#### MPS IVA

In MPS IVA, or Morquio A, the deficient activity of *N*-acetylgalactosamine-6-sulphatase (GALNS) causes accumulation of the GAGs KS and CS in multiple tissues with skeletal and connective tissue abnormalities [57].

The *GALNS* gene, encoding the lysosomal enzyme *N*-acetylgalactosamine-6-sulphatase (*GALNS*; E.C. 3.1.6.4; MIM #612222), maps to chromosome 16q24.3, has a length of about 50 kb, and is organized into 14 exons [58]. The *GALNS* cDNA is 2339 bp in length with a 1566-bp open reading frame encoding a 522-amino acid protein [58, 59]. The GALNS protein is found as a homodimer, but it has also been described in a multiprotein complex with other lysosomal enzymes [60].

To date, at least 333 different *GALNS* gene mutations causing Morquio A disease have been described (see the Human Gene Mutation Database [26]). Most of them are ‘private’, with only a few being common. Genotype-phenotype correlations have been reported for some of the recurrent mutations, representing founder alleles in certain population groups [61]. Thus, the p.Ile113Phe [62] and p.Thr312Ser missense variants recur in patients of British-Irish origin and a milder phenotype has been associated with the residual activity of the mutant p.Thr312Ser protein [63].

In the *GALNS* gene, large deletions and/or rearrangements and a uniparental disomy have also been reported [64–66]. Deep intronic mutations have also been detected (Morrone, personal data). So far, no pseudodeficiency has been described in the *GALNS* gene.

#### MPS IVB

The β-galactosidase gene (*GLB1*; E.C.3.2.1.23; MIM #611458) maps to chromosome 3p22.3, spans more than 60 kb, and is organized into 16 exons [67]. The *GLB1* gene gives rise to two alternately spliced mRNAs: a transcript of 2.5 kb encoding the lysosomal enzyme β-galactosidase and a transcript of 2.0 kb encoding the elastin-binding protein (EBP). Both GLB1 transcripts form protein complexes. β-galactosidase lysosomal enzyme or GLB1 is stabilised in a lysosomal multienzyme complex with protective protein/cathepsin A (PPCA), neuraminidase (NEU1), and *N*-acetyl galactosamine-6-sulphatase (GALNS) [60]. EBP is required for the assembly of tropoelastin monomers into elastic fibres on the cell surface where it forms a complex with PPCA and NEU1 [60, 68, 69].

Mutations in the *GLB1* gene are responsible for two allelic disorders: the neurodegenerative GM1 gangliosidosis (non-MPS) disorder (MIM 230500) and the rare MPS IVB (Morquio B) syndrome (MIM 253010) [70]. In contrast to GM1 gangliosidosis, patients with Morquio B retain neurological functions, but develop generalized skeletal dysplasia, keratan sulphaturia, and corneal clouding [71]. However, the clinical demarcation between GM1 and Morquio B at an early stage of diagnosis can be very difficult and obscured by a late-onset mental regression and by the presence of keratan sulphaturia in juvenile GM1 gangliosidosis forms.

The *GLB1* mutations underlying Morquio B syndrome affect the catabolism of KS but have little effect on GM1 gangliosides.

To date, 215 *GLB1* gene mutations have been reported (see the Human Gene Mutation Database [26]); among these, 22 were also identified in patients with Morquio B but only a few can be related to the specific phenotype [72–74]. The c.817_818del TG insCT leading to p.Trp273Leu is one of the most common mutations leading to a Morquio B phenotype [72].

Some *GLB1* mutations have been identified in both GM1 and Morquio B, and the same genetic assessment has been shown in patients who exhibited different symptoms, further complicating possible prognoses in these individuals [72–74]. The common p.Arg201His detected at the heterozygous level has been correlated with the juvenile GM phenotype but it has also been detected in a 15-year-old Morquio B patient harbouring the mutation at the homozygous level [73]. Thus, phenotypes in compound *GLB1* heterozygous genotypes sometimes remain difficult to predict [75, 76]. In addition, the *GLB1* gene polymorphic variants p.Arg521Cys, p.Ser532Gly, and p.Arg595Trp have been reported to be associated with beta-galactosidase enzymatic pseudodeficiency [77].

#### MPS VI

The *N*-acetylgalactosamine 4-sulphatase (arylsulphatase B) gene (*ARSB*; MIM #611542), located in 5q14.1, spans a region of about 206 kb and contains 8 exons encoding a 533-amino acid glycoprotein [78]. ARSB deficit causes a pathological accumulation of undegraded DS in most organ systems. Presently, 197 different alterations have been reported, the vast majority of which are missense/nonsense lesions (Table 2). Since no common mutations have been reported up to now, the potential for studies of genotype-phenotype correlation in MPS VI is somewhat limited. Only one pseudodeficiency allele was reported a few years ago, p.Thr212Ile, in a family for which very low ARSB enzyme activity was described in relation to normal clinical findings [79]. Interestingly, the p.Ser384Asn (S384 N), one of the most common polymorphisms of the gene [80], had been traded for several years as a pathogenetic variant and correlated with a severe phenotype, leading to a series of incorrect molecular diagnoses of the ARSB gene [81].

#### MPS VII

The gene encoding β-glucuronidase (*GUSB*; MIM #611499) is 21 kb long and contains 12 exons. Two types of cDNAs arise through alternative splicing, resulting in an exon 6 corresponding to the 153-bp deletion in the shorter of the two types [82]. The presence of unprocessed multiple pseudogenes requires particular attention in diagnostic mutation analysis. To date, 64 different mutations have been reported in the *GUSB* gene (Table 2). Another complication reported in MPS VII diagnosis is the presence of the missense polymorphism p.Asp152Asn producing a pseudodeficiency allele that leads to greatly reduced levels of beta-glucuronidase activity (27% of the control) without apparent deleterious consequences [83].

#### MPS IX

The gene encoding hyaluronidase (*HYAL1*; MIM #607071) contains 3 exons and spans 3.5 kb [84]. *HYAL1*, together with *HYAL2* and *HYAL3*, constitutes a multigene family for lysosomal hyaluronidase. To date, only one patient in the original report, and three others belonging to a second family have been described, yielding a total of three different mutations responsible for the disease (Table 2) [85, 86].

## Conclusions

Although MPS share many clinical features among different enzyme deficiencies, they can also show a wide spectrum of clinical severity within each enzyme deficiency. Hence, even in the presence of typical clinical signs and symptoms, the choice of samples and diagnostic tests can be different for each MPS and require a multidisciplinary approach, including laboratory specialists and clinicians.

Increased urinary excretion of GAGs may only support the clinical suspicion of MPS and it is necessary to follow this with specific genetic testing including enzymatic and/or molecular analyses, performed on suitable samples, as leukocytes and/or cultured cell lines. While both analyses must be considered complementary for the definitive diagnosis of MPS and the genetic counselling, the molecular analysis is essential for carrier detection, and can sometimes predict prognosis and support therapeutic choices. The recently proposed NGS techniques will very soon complement the conventional testing, in particular for those patients where a differential diagnosis is difficult to perform.

## Abbreviations

CS: Chondroitin sulphate
DBS: Dried blood spot
DS: Dermatan sulphate
GAG: Glycosaminoglycan
HS: Heparan sulphate
KS: Keratan sulphate
MPS: Mucopolysaccharidosi(e)s
MS: Mass spectrometry
NBS: New-born screening
NGS: Next-generation sequencing
Pd: Pseudodeficiency
